# Freedom for some, but not for Mum: the reproductive injustice associated with pandemic ‘Freedom Day’ for perinatal women in the United Kingdom

**DOI:** 10.3389/fpubh.2024.1389702

**Published:** 2024-08-07

**Authors:** Sergio A. Silverio, Elizabeth J. Harris, Leanne Jackson, Victoria Fallon, The PRaM Study Group, Laura K. Soulsby, Paul Christiansen, Leonardo De Pascalis, Joanne A. Harrold, Abigail Easter, Peter von Dadelszen, Davor Jurković, Laura A. Magee

**Affiliations:** ^1^Department of Psychology, University of Liverpool, Liverpool, United Kingdom; ^2^Department of Women & Children’s Health, King’s College London, London, United Kingdom; ^3^Gynaecology Diagnostic and Treatment Unit, University College London Hospitals NHS Foundation Trust, London, United Kingdom

**Keywords:** COVID-19, reproductive justice, pregnancy, postpartum period, perinatal period, motherhood, women’s health

## Abstract

**Introduction:**

Healthcare services for pregnant and postpartum (‘perinatal’) women were reconfigured significantly at the advent and for the duration of the SARS-CoV-2 pandemic, and despite the United Kingdom announcing ‘Freedom Day’ on 19 July 2021 (whereafter all legal lockdown-related restrictions were lifted), restrictions to maternity (antenatal, intrapartum, and postnatal) services remained. This study presents data from eight perinatal women about their experiences of psychosocial wellbeing and maternity care in the post-‘Freedom Day’ epoch.

**Methods:**

Semi-structured interviews were conducted virtually, with data recorded, transcribed, and analysed by hand. Grounded theory analysis was employed with the final theory assessing the reproductive injustice of the pandemic ‘Freedom Day’.

**Results:**

Analysing iteratively and inductively led to four emergent themes: ‘A Failing System, Failing Women’; ‘Harm Caused by a State of Difference’; ‘The Privileges (Not Rights) of Reproductive Autonomy, Agency, and Advocacy’; and ‘Worried Women and Marginalised Mothers’. Together, these themes form the theory of ‘Freedom for some, but not for Mum’.

**Discussion:**

Women experienced a lack of high-quality reliable information about the pandemic, vaccination against the virus, and the changes to, and decision-making surrounding, their perinatal care. Women recognised healthcare professionals and maternity services were stretched and that maternity services were failing but often reported hostility from staff and abandonment at times when they were unsure about how to navigate their care. The most singular injustice was the disparity between women having to accept continuing restrictions to their freedom whilst receiving maternity care and the (reckless) freedom being enacted by the general public.

## 1 Introduction

The novel coronavirus, SARS-CoV-2, or ‘COVID-19’ spreads quickly across the globe, gripping the UK in January 2020, and taking hold of both the population and healthcare systems. The World Health Organization (WHO) announced the pandemic, with SARS-CoV-2 being a public health emergency of international concern (PHEIC) status on 11 March 2020. After 3 years, 3 months, and 5 days (5 May 2023), the pandemic was downgraded from being a global health emergency (1). In response to the pandemic, the UK Government issued national lockdown restrictions, comprising mandated ‘stay-at-home’ orders (2), and a range of physical and social distancing measures (including the closure of much of the service industry and ‘non-essential’ shopping). In addition, they recommended ‘shielding’ for those deemed to be most vulnerable to infection, which initially included pregnant and postpartum women (3). In parallel, the healthcare system was substantially reconfigured to allow for the redirection of the workforce to ‘COVID wards’ (4). This resulted in substantive reconfigurations to maternity services, as guidance evolved rapidly regarding the provision of care for both pregnant and postpartum women (3, 5). Within maternity care, face-to-face antenatal care was restricted, and postnatal care was nearly withdrawn in its entirety (4, 6, 7). This was a dramatic adjustment as regular antenatal visits and surveillance (e.g., blood tests and ultrasound) have been established as practices to optimise pregnancy outcomes. This abrupt change in guidance provoked inevitable anxiety amongst perinatal women (8). In addition, women were forced to access care without their partner or chosen birth companion (9, 10), and shortly after birth as partners were forced to leave the maternity setting (11). These changes directly contradicted WHO recommendations which encourage pregnant women to be accompanied by a support person, who poses no threat to labouring women, their babies, other patients, or healthcare professionals (8).

Sparse information regarding the risks of contracting the virus during pregnancy caused further avoidable distress amongst pregnant women (10, 12), highlighting the importance of clear information to ensure perinatal autonomy is safeguarded (13). The UK population experienced varying levels of national, regional, and local lockdown restrictions between 31 January 2020 (when the first COVID-19 positive case had been recorded in the UK) and 19 July 2021 (whereafter all legal restrictions relating to lockdowns were lifted)—the latter date colloquially known as: ‘Freedom Day’ (14). Many feared a significant third wave of hospitalisations and deaths (15), and hospitals therefore did not return to pre-pandemic ways of delivering their services—especially true within maternity care which still placed restrictions on partner presence, levels of face-to-face appointments, and postnatal care provision (5, 16). Therefore, there was discordance across the UK, whereby society returned to a level of para-pandemic normality post-‘Freedom Day’ (17), whilst perinatal women continued to face restrictions to their care (3). Losing sight of woman-centric priorities and failing to put women and children first throughout such crises may have substantial, if not staggering, negative impacts in the future (8, 18).

To date, much of the research conducted into the experiences of pregnant and postpartum women during the pandemic has focussed on the early stages of the pandemic (6, 7, 19–23); the psychological wellbeing and mental health consequences of contracting the virus and/or pandemic-related lockdown restrictions (24–28); vaccine hesitancy throughout the perinatal period (29–34); or the consequences of reconfiguring of maternity care and perinatal mental health services (5, 35–39), rather than the residual effects for perinatal women in the post-‘Freedom Day’ epoch.

Therefore, this study aimed to explore the psychosocial experiences of women navigating pregnancy and the postnatal period in the time after the so-called pandemic ‘Freedom Day’.

## 2 Methods

### 2.1 The present study and the study team

The present analysis was part of a larger study called ‘The Pregnancy and Motherhood (PRaM) during COVID-19 Study’. The study recruited pre- and post-natal women to semi-structured interviews via a national on-line survey about their experiences of mental health, psychosocial wellbeing, and perinatal care during the COVID-19 pandemic. The present analysis employs a classical grounded theory analysis methodology (40). The authors are a cross-disciplinary team of researchers and clinical academics, with expertise in perinatal psychology (SAS, LJ, VF, AE, and EJH), women’s mental health (SAS and AE), maternity care (LAM and PvD), gynaecology (DJ), and maternity health services delivery (SAS, AE, LAM, PvD, and DJ). Data analysis was led by one researcher, experienced in qualitative research with particular expertise in sensitive interviewing (SAS), supported by two others (EJH and LJ), with a background in Psychology. Regular meetings were held to discuss emergent themes and the developing theory, and the final analysis was discussed with the wider team. Researchers and analysts utilised bracketing (41) to ensure no *a priori* assumptions about the population (women), phenomenon (pregnancy and postpartum period), and context (post-pandemic ‘Freedom Day’ in the UK) were carried through into the results.

### 2.2 Theoretical perspective

For the purposes of this analysis, we adopted a theoretical perspective in-line with gendered lifecourse research (42–44), meaning, in our study setting (the UK—a Western society), the ‘normative’ lifecourse for women includes pregnancy and childbirth. As the pregnancy and postpartum period is transitionary both physiologically and societally, it offers the opportunity for a site of empirical inquiry. A theoretical perspective based around lifecourse analysis is acceptable and harmonious for use with grounded theory analysis, given its endeavour to understand the distinct trajectories of people’s lives which are demarcated by positive transitions and negative ruptures (40). Therefore, critical realist ontological and objectivist epistemological philosophies underpin our study (45). We regarded our positionality to have a critical approach to reflexivity and an objective outsider position within the data (as none of the study team were themselves pregnant or postpartum at the time of the study). In summary, the research can be seated in a post-positivist paradigm (45), whereby the narratives of participants are accepted as ‘truths’ or ‘lived realities’ even if recalled incorrectly, tarnished, embellished, or gilded (46), as the act of the acquisition of (even false) knowledge itself brings us closer to the truth of the reality experienced.

### 2.3 Ethics

Ethics approvals were sought and granted from the University of Liverpool Research Ethics Committee on 7 April 2020 (ref: IPHS/7630). Participants had consented to be contacted to take part in interviews during the online survey, and separate consent was requested to participate in the interviews. Participants were made aware of their right to withdraw and were fully debriefed after interviewing.

### 2.4 Recruitment, setting, and participants

Pregnant and postpartum women who completed the PRaM Study online survey [see (25)] were redirected to a secondary Qualtrics survey, which allowed women to provide their telephone number and e-mail address, to be contacted for participation in semi-structured, telephone, interviews about their pre- or postnatal experiences, post-‘Freedom Day’. Electronic consent forms were signed; however, audio-recorded verbal consent was also taken before each interview to ensure the participant was still happy to take part in the current study.

Eligibility criteria were consistent for the PRaM Study online survey which had three waves of data collection [see (25)]: maternal age above 18 years, third trimester of pregnancy or postnatal, English-speaking, and UK resident. Qualitative findings from the first wave (‘introduction of social distancing’: 23 March 2020 onwards) and second wave (‘initial easing of social distancing restrictions’:11 May 2020 onwards) of data collection within the broader PRaM Study can be found elsewhere (6, 47–49). Recruitment for the third wave commenced approximately 30 days after ‘Freedom Day’ (19 July 2021), allowing for a ‘washout period’ preventing contamination of extant policy and practices which may not have been changed with immediate effect, and ensuring women could be oriented to their current experiences at the time of interview.

Participants (*N* = 8; provided with pseudonyms) were either pregnant (*n* = 4: Violet, Hetty, Maeve, and Marta) or postpartum (*n* = 4; Iris, Grace, Helena, and Wilma), ranging in age from 24 to 39 years (M_Age_ = 31.5 years). The majority were educated to at least an undergraduate degree level (*n* = 6) with most having Master’s degrees (*n* = 4) and the remaining participants having attained A-Levels (*n* = 1) or Post-Secondary School Qualifications (*n* = 1). Most participants were employed (*n* = 6) in a range of professions (Account Manager, Doctor/Public Health, Chief Executive Officer, Customer Services) and/or were in higher education (*n* = 2), with the remaining participant reporting themselves as a homemaker (*n* = 1). The pregnant women were between 30 and 37 weeks of gestation (M = 34 weeks), and postpartum women had babies ranging from 2 to 16 weeks old (M = 8.5 weeks).

### 2.5 Data collection

Interviews were semi-structured (50), with the interview schedule having been created by members of the PRaM Study team with expertise in perinatal mental health. The interview schedule followed a chronological structure to enable for the full perinatal period (i.e., pregnancy and postpartum period, as appropriate) to be discussed, with enough flexibility for interesting or pertinent discussions to be probed and followed up with further spontaneous questions. Interviews were conducted via telephone or video-conferencing software (as per participants’ preferences) at a time and on a date convenient to the participant. Data collection spanned from October to December 2021. Interviews lasted between 48 and 86 min (M_Time_ = 67 min). Data collection followed guidelines for best practice when conducting interviews of a sensitive, challenging, or difficult nature (51), meaning field researchers had regular supportive supervision and checked-in before and after interviews were conducted. Interviews were recorded, transcribed intelligently (i.e., omitting some of the contextual matter of verbatim transcription), and formatted in Microsoft Word for electronic ‘hand’-coding. All participants were reimbursed £10 for their time and provided with a full debrief.

### 2.6 Data analysis

A classical grounded theory analysis (40) was employed allowing for inductive and iterative work to be undertaken with the data to derive codes, super-categories, themes, and the eventual theory (52). Grounded theory analysis follows seven key principles (40, 53): (i) No *a priori* assumptions; (ii) Data-driven analysis; (iii) *In vivo* coding; (iv) Constant comparison; (v) Reflexive practice; (vi) Theoretical sampling; (vii) Developing a testable theory.

This cross-disciplinary approach to classical grounded theory has nine study phases (52): Study Design and Development; Preparing Data; Cleaning Data; Coding; Theme Development; Theory Generation; Defence of Theory; Writing-up; and Testing the Theory; each of which have several data handling stages, totalling twenty. In this analysis, we had initially thought to separate the two groups of women (i.e., pregnant; postpartum), thereby facilitating comparison between the groups after analyses were complete. As it became evident quickly that data were saturating across the two groups, the population of interest became a combined group of perinatal women. Saturation was measured on two axes: data (whereby recruitment could be stopped as similar thematic narratives were being derived from interviews) and theoretical (whereby analysis could be stopped as derived themes were adequately supported by data and all avenues of potential themes were exhausted). Data and theoretical saturation were achieved at eight and six participants, respectively. These levels of saturation with relatively few participants are achievable in studies with such specific parameters for population, phenomenon, and context (54).

Data were analysed in-line with established principles of grounded theory, meaning two passes of coding (EJH, LJ, and SAS): ‘open’ (using verbatim data as initial codes for each line or sentence of the data) and ‘focussed’ (using broader and more descriptive codes to represent wider trends in the dataset). ‘Super-categories’ (lower-order themes) were then formed by merging, fragmenting, and re-arranging focussed codes before they themselves were either collapsed, split, and further ordered to develop (higher-order) ‘themes’ (SAS, EJH, and LJ). The final analytical result—a theory—was derived by assessing the relationship between these themes (SAS) (52, 53). Code, super-category, theme, and theory development were consultative; with defence of each stage occurring between members of the analytic team (SAS, EJH, and LJ), thus improving overall credibility and rigour.

## 3 Analysis and findings

**Figure 1:**
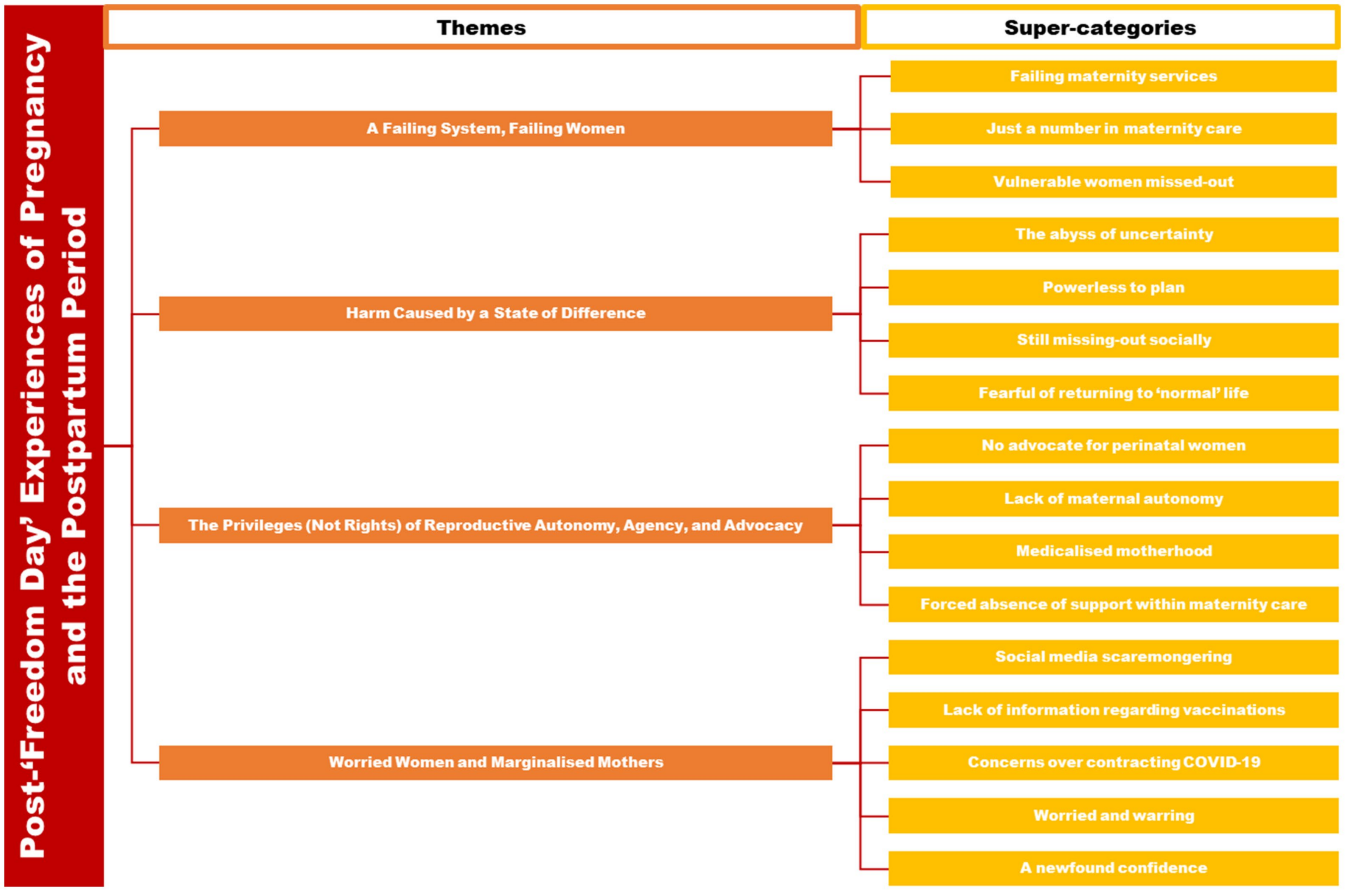
Initial thematic diagram of the themes and super-categories.

Grounded theory analysis rendered four emergent themes (see Figure 1): ‘A Failing System, Failing Women’ (with super-categories of: Failing maternity services; Just a number in maternity care; Vulnerable women missed-out); ‘Harm Caused by a State of Difference’ (with super-categories of: The abyss of uncertainty; Powerless to plan; Still missing-out socially; Fearful of returning to ‘normal’ life); ‘The Privileges (Not Rights) of Reproductive Autonomy, Agency, and Advocacy’ (with super-categories of: No advocate for perinatal women; Lack of maternal autonomy; Medicalised motherhood; Forced absence of support within maternity care); and ‘Worried Women and Marginalised Mothers’ (with super-categories of: Social media scaremongering; Lack of information regarding vaccinations; Concerns over contracting COVID-19; Worried and warring; A newfound confidence). Themes are presented below, with the most eloquent quotations chosen to illustrate each theme. Supplementary quotations can be found in Table 1.

**Table 1:** Supplementary quotations.

A Failing System, Failing Women	Harm Caused by a State of Difference	The Privileges (Not Rights) of Reproductive Autonomy, Agency, and Advocacy	Worried Women and Marginalised Mothers
*‘I’ve not needed anything else. But I think, the support, as in doctors and the NHS, it was terrible’.* (Grace)	*‘I think it would be hard, for example, if my little girl’s nursery closed, that would be something that might stress me out a bit, having both children at home, and my husband working full-time’. ‘…So that is a bit of a worry, but again, there’s not really a lot we can do about it, so it’s just about dealing with it as it comes’.* (Marta)	*‘I suppose that’s probably a backlog from COVID really, of non-essential things but if it’s impacting on feeding it is quite essential for newborns. But again, that was something that wasn’t a big issue for us because we paid privately but again, it feels unfair if you cannot afford it that you just have to wait’.* (Helena)	*‘More information, better guidance, better advice. I think about other people who aren’t as kind of research-y as me. And they are just without the advice And I do not think people should be having to be going to Facebook for the advice on something that actually could be really important to your unborn child’.* (Marta)
	*‘Yes, I think if we go into restrictions again that is going to be probably the biggest impact, is not being able to have that support of friends and family as on-hand, and with your appointments being virtual, or phone, I do think it really helps to have face-to-face appointments’.* (Marta)		
	*‘We do not really feel as capable of making long-term plans anymore because we just do not trust things to not change dramatically’.* (Violet)		
*‘I have not really had any advice from the midwife. But she did make a note in my file that she’s talked to me about breastfeeding, but she did not. But then the midwife experience has not been brilliant in my area, because I’ve seen a different person every time’.* (Marta)	*‘…we still cannot really plan particularly a home birth and we have been preparing for one, but having the birth pool and everything we have been setting up at home, we cannot really… we are not even how sure that we are going to be able to use it because no one really knows what is happening and when people can do what they want most of the time, it does not really give you much faith that things are going to change for the better’.* (Violet)	*‘I think having a baby this side of the pandemic has been easier than those who had babies at the beginning of the pandemic when nobody knew what they were doing. Healthcare-wise, people were not seeing health visitors, and men or birthing partners were not allowed in the room until active labour; that would have been really fucking hard – sorry’.* (Wilma)	*‘So yes, I think if things like social classes stop, then that will make it a different maternity experience… I do think it’s good to have people in your social circle that have got children the same age as you, so that you can rant and moan when they are not sleeping or get advice about different things’.* (Marta)
*‘She was trying to breastfeed, and then the hospital rang her and said she’d tested positive for COVID, so then she could not get any help for 10 days. And nobody would come to the house, nobody would see the baby to cut his tongue-tie, and then she ended up having to go on to formula, because she just could not, it just wasn’t working. And I think things like that are a shame, that they cannot put the things in place that even if you had COVID or the baby had COVID, that you could still help them’.* (Marta)	*‘I do not know that because the health visitor was like ‘Well, there’s nothing available’, or ‘There’d be this breastfeeding group, but you cannot go because it’s not available’. So, there are still things that are restricted, it seems like, or that have not been reinstated, that would probably be really beneficial’.* (Wilma)	*‘I think make it clearer what support groups are still available, be that volunteer support groups, breastfeeding groups or whatever, because I think that’s certainly something that I’ve seen a lot of on social media, where new mums are really struggling with breastfeeding and aren’t in an area where there’s an Infant Feeding Team and they have really struggled to get support’* (Wilma)	

### 3.1 A Failing System, Failing Women

The first theme in this analysis provides women’s perception of the context of maternity care and the perinatal healthcare system in the United Kingdom. To women in this study, these were seen as failing—not necessarily the fault of the healthcare system or healthcare professionals working within it—but nonetheless failing, and in doing so, subsequently failing the women in its care:

*‘…I completely understand that the NHS is totally overwhelmed with what they have got going on already. But yes, I think just general concerns, just from people that I speak to as well, is that you are just another number at the moment, everybody’s just trying to get through the backlog, as it is. So, you are in and out’* (Maeve).

*‘…now that pregnant women are one of the highest groups on ventilators in the wards at the moment, I think there’s just been a really missed opportunity to get the right information out to pregnant women’* (Marta).

In addition, many raised concerns about the pandemic circumstances, which included an increase in virtual and a reduction in face-to-face care, might leave women living with social complexity (e.g., severe mental health, intoxicated or abusive households, and high levels of deprivation) and those struggling during pregnancy and soon thereafter, not being detected for physical and/or mental health concerns, with potential maternal, foetal, and newborn risks:

*‘I just feel like I’m not vulnerable, and if I was, I just think there’s potential missed opportunities with things being virtual…there could just be missed opportunities to pick things up. Especially around mental health and wellbeing’* (Marta).

In interviews, women recalled how palpable the demands on staff were and how stretched they had become during the pandemic, and although sympathetic, women often pondered how this might affect the care they would receive or recalled how detrimental the shortage of staff was to their care, sometimes with women having to look after themselves whilst in recovery:

*‘The staff on the ward and the midwives were really busy so it was difficult to get them to help you, so I’m just slightly concerned that if there’s no visitors there to help you, it might make things more difficult’* (Hetty).

*‘We were left alone in a side room from half-seven at night until half-four in the morning with a five-day old baby and I was five days post section. It wasn’t about me, but there wasn’t a bed there for me to lie down, it was just a hardback chair, and I was still bleeding, and my scar was still essentially still an open wound at five days’* (Wilma).

As well as not being cared for appropriately, many women reported incidences of incivility from healthcare professionals:

*‘So, I literally called them straight back and she was like ‘No, you have missed your appointment’. And I was like, ‘Well it was an hour-long appointment, I called you at one minute past two, how is it that there’s now no longer time to have my appointment? It just did not make sense, because that time was set aside for me, and I called you back one minute later’. It just seemed like I was palmed off a little bit’* (Iris).

Dissatisfaction with maternity care was apparent through all interviews as women frequently complained about the attitudes of some healthcare professionals, as well as pressures on the healthcare system inhibiting development of trustworthy relationships between women and staff where women felt they could disclose their concerns:

*‘You never see the same person twice……… You just feel like they want you in and out. If I was suffering from depression, for example, and I really needed to talk to someone, I do not really feel like there’s the opportunity, or the trust, because you have not built a relationship with anyone’* (Marta).

There was a general sense from women that it was not always actions which failed them, but sometimes also the circumstances which led to them being alone and feeling, at best, unsupported, and, at worst, abandoned:

*‘When I miscarried, that was in May last year. At that point, no-one could go to the hospital with you so I had to do that on my own, and that was very difficult because that was a time when I would have really appreciated being able to have my partner there with me’* (Helena).

*‘So why now is it not needed? Does that make sense? Like, if you were checking the baby’s development regularly and checking that everything’s okay, why now because we have got a pandemic has that changed? Because the baby is still growing, and you still need to tell me or check that he’s doing alright’* (Grace).

### 3.2 Harm Caused by a State of Difference

Many women struggled to remain hopeful about their birthing and postpartum experiences. This was especially with regard to them involving or incorporating their own choices and decisions as they were concerned about not having their agency and decision-making accommodated. This was exacerbated by the fact that hospital-based restrictions remained, and frequently changed, despite the remainder of society enjoying ‘Freedom Day’ and beyond without any restrictions.

*‘I have asked outright how realistic it is to actually plan for that and no-one is willing to give me an answer. They just said, ‘It might be wise to make two plans’, so, in the back of your mind, expect not to have the experience you want’* (Violet).

The so-called ‘Freedom Day’ did not appear to change the uncertainty felt by many perinatal women about planning for the arrival of their baby and postpartum life but, if anything, appeared to exponentially increase feelings of anxiety at the thought of government restrictions and protective practices being removed:

*‘But what scared me was the idea that people were being let loose, and there were people who had chosen not to get the vaccine for whatever their reasons are, but there were people living without restrictions when I was in my most vulnerable point of pregnancy, and that terrified me. So, for me, it wasn’t Freedom Day, for me it felt like even more of an isolation day’* (Wilma).

*‘Yeah, it just has a much worse impact on our ability to prepare for anything. And every time something is cancelled, we know that it is because rates [of COVID-19] have gone up, and you just feel powerless in being able to do anything about it’* (Violet).

Despite the easing of all legal restrictions on social and physical distancing, women interviewed post-‘Freedom Day’ reported they were still missing out socially and felt disconnected from their social support networks:

*‘I think the one thing that has not opened yet – or at least has not fully-fully – was children’s centres… I always imagined that that would be a great source of support’* (Helena).

*‘So, you just feel like you have missed the opportunity to meet people that you could spend the next year of your life with, with babies and going out and about and that bonding that you do when you are pregnant, and then have your babies afterwards’* (Maeve).

Therefore, in a sense, ‘Freedom Day’ bypassed perinatal women and was perceived as a day marking further isolation for perinatal women, in contrast to the rest of the population who were free to live without restrictions:

*‘It made us anxious because [when ‘Freedom Day’ came]… knowing that the things most likely to protect us were just suddenly being removed probably made that worse’* (Violet).

*‘… we were getting out a little bit more and did not really feel like ‘Freedom Day’ made a huge difference’* (Marta).

The perception that economically viable aspects of life were returning to normal opening and operating hours and circumstances, even when parts of healthcare—such as maternity services, which were essential for physical health and mental wellbeing—remained ‘off limits’ to the same sense of normality:

*‘Although restrictions have been lifted, it’s been difficult to actually find baby groups that are still going. They’re still saying that because of the government guidance, even though obviously restrictions have been lifted for a while, but that seems to be on every website. There does not seem to be any that are open unless it’s the ones that you have to pay for’* (Iris).

This further entrenched socio-economic inequalities by restricting accessibility to holistic maternity care and support to those affluent enough to fund themselves, reducing the sense of community amongst pregnant women and birthing people which would otherwise be facilitated by antenatal classes or mother-and-baby groups:

*‘I think [pause] community care is really important because you do have a lot of people who do feel isolated… the health visitor was here, she was like ‘Oh well, normally there’d be this, but that is not available to you now because of the pandemic’, so I think there are a lot of things, like [sigh] the community aspect that seems to have gone’* (Wilma).

There was the sense that the phasing out of lockdown restrictions resulting in ‘Freedom Day’ was ‘too much, too soon’ and came at the expense of those who required maternity services, care, and support:

*‘Yeah, it just felt very all or nothing, without an in between or phasing out. So, yeah, it was a bit anxiety-provoking… it did feel just too much too soon, and I was not comfortable going back to anything baby-related to pre-COVID days for a while’* (Violet).

In summary, there was a disparity between the population experiencing ‘Freedom Day’ and perinatal women who were not experiencing the same lack of restrictions. In fact, it was the disparity and difference between perinatal women’s and other people’s lives and the perceived indifference towards perinatal women’s plight during this time by those who had brought in the new rules, which caused most anger, frustration, and harm to psychosocial wellbeing:

*‘…it’s pretty infuriating sometimes when you think about what you sacrificed for the sake of what these people have told you is the safest thing for us all to be doing, which is completely standard and fair enough. But when you see that they have not done the same thing, and have not made the same sacrifices, I think it’s pretty gut wrenching’* (Maeve).

### 3.3 The Privileges (Not Rights) of Reproductive Autonomy, Agency, and Advocacy

The perinatal women in this study frequently expressed that they either did not feel listened to or even seen by healthcare professionals and, therefore, did not feel in control of, or supported during, their maternity journeys:

*‘You just did not really have a choice, and I think that’s probably what would happen again for the next one. You feel like you would want to take things into your control, and I think not having a voice and feeling like you cannot be listened to, all of those kinds of things, is really difficult, because you want the Government to hear how you feel, personally’* (Maeve).

As a result, women felt no one was advocating for them at a time when they believed they needed it most:

*‘And I guess there’s no one voice for pregnant women, to advocate for us, and say, ‘Oh yes, you should, or you should not’. And then you think somebody should take that upon themselves to be clear about it’* (Maeve).

The need for advocacy during the perinatal period was particularly apparent due to the restrictions around intimate or chosen birthing partners being allowed into the healthcare spaces:

*‘…my husband will be allowed in for the C-section and then he’ll be allowed to stay for two hours immediately afterwards, this is providing that we are both negative for COVID. So, you have to be swabbed before, and then he would be allowed in the following day for two hours’* (Hetty).

*‘I am aware that I can have my birth partner with me as well, but I know that is something that has been withdrawn quite quickly as well’* (Violet).

This, unfortunately and rather traumatically, extended to situations where women had concerns about their pregnancy, where women reported having to wait for reassurance scans and results on their own:

*‘…before I had my early pregnancy scan, my partner could not sit with me in urgent care for the screening…that was quite stressful because this thing we were planning for had finally happened and then knowing that we might have lost the baby, and I would have had to essentially deal with that by myself was really hard’* (Violet).

Whilst women struggled with the concept of the right to their chosen and consented birthing partner possibly being withdrawn at any moment, they also reported that even when partners were present, this was never allowed to be for very long even in the context of the post-‘Freedom Day’ period:

*‘Then I delivered within a couple of hours and once I was delivered, the midwife was like, ‘Okay, as soon as you are ready, you need to move onto the ward’. And I was like, ‘Okay, we’ll just pack our stuff and I’ll see the baby and then we’ll move onto the ward’. Everything felt rushed……… And then once I went onto the ward, my husband wasn’t allowed on the ward with me’* (Grace).

*‘I was just so distressed, because the thought of having to, through COVID, being stuck in a little room on my own post-section trying to care for her and myself, it was just too much, so that was horrific in the first few days after birth’* (Wilma).

Ultimately, women believed they had to navigate the perinatal period alone when left feeling that healthcare professionals were not advocating for them and lacking the presence of chosen and consented birth partners. This often led women to believe their reproductive autonomy had been compromised, and they were left without agency, frustrated by the outside world living free of rules, whilst their perinatal period was still defiantly ‘locked down’:

*‘…you just constantly hear stories of people being told, ‘You cannot have the birth that you want’, whether it is a midwife-led unit or a home birth because the resources are all being used up, so it is a little frustrating sometimes when that is quite important things for me. I would like to be at home’* (Violet).

### 3.4 Worried Women and Marginalised Mothers

In what is the dominant theme of this analysis, participating perinatal women reported the perception that there was a general lack of reliable information distributed. This resulted in many women turning to social media for answers. This led to further issues given that many women found the information they retrieved on social media being just as inaccurate if not more so, having the effect of scaremongering, rather than alleviating concerns:

*‘No, there really is not a single source of information. And Google is not the place for pregnant women to be searching for their antenatal information. That should be coming from one source that is trustworthy… And it would take away a lot of the anxiety and the unknowns around pregnancy, which is hard enough in a normal pregnancy in normal times, let alone in a pandemic’* (Maeve).

Even at this stage of the pandemic, women reported an absence of information for pregnant and postpartum women about contracting the virus, what they should do, and what impact it might have on them and their babies:

*‘I’d rather be prepared than not. They should, maybe, when you are at the hospital, they should just say to you, ‘Listen…’ You know like they give you information leaflets about sepsis and meningitis, and all this, in babies, why not give you one for COVID?’* (Grace).

With women feeling compelled to obtain their own information, there was concern whether or not all women were able to access credible information, and if they did, whether they could understand its meaning in full given the various unknowns which were extant about the pandemic:

*‘There are people who aren’t in my position, who will not understand that information that’s available, who will get really confused by it and need more guidance, and I just think there needed to be more decisive guidance rather than ‘Oh, just go and read what’s on this website’, because people will not understand it, it wasn’t written in layman’s terms really’* (Wilma).

What perhaps generated most concern amongst women in this study was that even after ‘Freedom Day’, there remained an absence of advice regarding the protocol for going into hospital to give birth, leading to the potential reality of the birthing experience remaining unknown:

*‘But since ‘Freedom Day’… there’s been no COVID advice whatsoever… I’ve not been given any information whatsoever about visiting times, hospital stays, whether you are likely to be in more, whether you are likely to be in less, what happens if you get COVID at this point… I mean obviously if you go into labour and you had COVID, you must still have to go to the hospital, so how does that work? What’s the protocol?’* (Maeve).

Women lacked confidence in the information they received about vaccination against COVID-19 and even doubted the information their received from healthcare professionals who they felt were unable to provide them with reliable information on whether the vaccinations during pregnancy were safe:

*‘I think that they definitely should give more advice on the vaccine. And actually, had some advice to give, you know if you ask for it from a midwife, and they had nothing to give me apart from, ‘Google it’. And yes, nobody’s talked to me about what the impact of a vaccination would have on feeding either’* (Marta).

Much of this doubt came from women’s perception that the Government took too long to distribute vaccination advice to pregnant and postpartum women, who should have been a priority group, as they had been in other nations:

*‘Yes, so clear vaccination advice from day one. It should not have taken them six months to tell pregnant women to actively get vaccinated…Because they left a lot of women uncertain, and probably quite scared, because we were not sure what we were basing the information on’* (Maeve).

In addition, women expressed both concern and genuine anxiety when the Government advice changed from equivocating about vaccination against COVID-19 for pregnant women to partial and, ultimately, full endorsement with pregnant and postpartum women being actively encouraged to have the vaccine:

*‘It was only when I was already about 26+ weeks that they said pregnant women could have it, so I think that change in guidance is still having an effect on pregnant women not getting the vaccine now. Because somewhere in your head, ‘Oh, they did not think it was safe and now they do, what happened?’* (Helena).

*‘So, then I ended up getting the AstraZeneca one, but ultimately then the advice came out, pregnant women should not get the AstraZeneca one. So, then you wonder, ‘Oh god, should I have done that in the first place?’* (Maeve).

Borne out of their frustration with how perinatal women were being handled by healthcare professionals, healthcare services, and the Government and governing bodies, many women reported one positive to come from this adversity, in that they could turn their worry into self-advocacy and fight confidently to take control of their perinatal journey and advocate for their babies during the unprecedented pandemic circumstances:

*‘And maybe that was because I was quite clear on my decision, I wasn’t going to people for advice, I’d already made my decision and said, ‘I’ve read the articles, the risk of getting COVID from 28 weeks is a much more profound impact on the baby than the unknowns of what the vaccine is’. So maybe I did not give people the opportunity to have an opinion on whether I should have had it or not’* (Marta).

When grasping for even the bare minimum advice, information, and care, women considered it their duty to speak-up and, on occasion, be demanding if they wanted to capture healthcare professionals’ attention:

*‘I think just be pushy with medical and your GP and all of that kind of stuff, to get the right information from them. Do not feel like you have to be quiet or be silent or anything like that. Push to get the right information for themselves’* (Maeve).

This newly found confidence meant women often reported feeling more in control and more aware of the potential outcomes for themselves and their babies:

*‘…there was a definite difference before and after I found out I was pregnant. I just became a lot more forceful in my boundaries’* (Violet).

*‘I feel a lot more confident advocating for her and advocating for myself now that she’s here, [pause] because I feel like there’s an expectation for mums to be like… Not necessarily to be like that, but to have some rules about how they want people to interact with their kids and stuff’* (Wilma).

### 3.5 Interpretation of the theory: Freedom for some, but not for Mum

**Figure 2:**
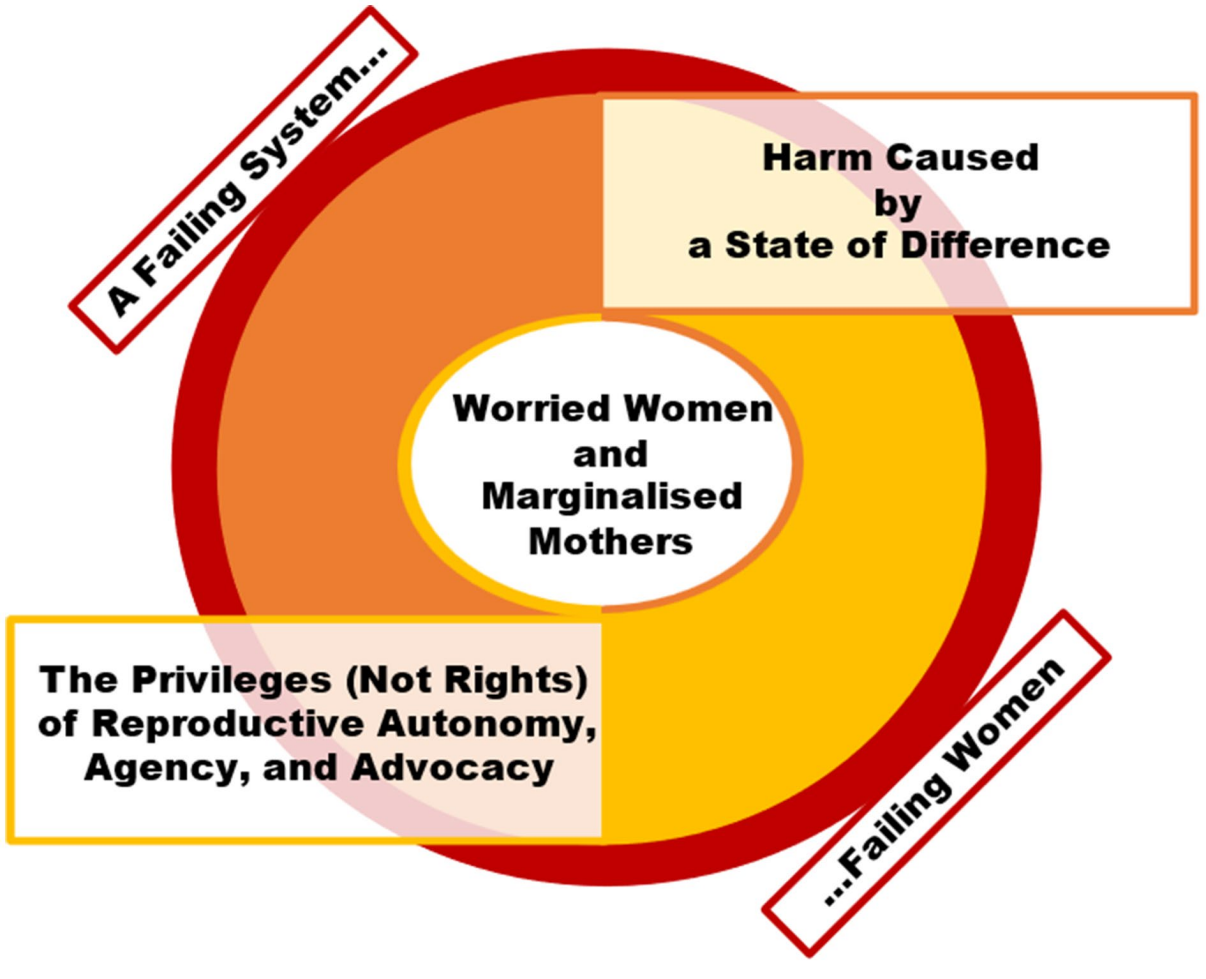
Final thematic diagram of the theory: ‘Freedom for some, but not for Mum’.

Together, the interaction of these themes demonstrated processional, cyclical, and causal relationships (53). Theme 2 (‘Harm Caused by a State of Difference’) and Theme 3 (‘The Privileges (Not Rights) of Reproductive Autonomy, Agency, and Advocacy’) exacerbated one another; the more freedom was given to citizens outside of maternity care, the more women felt their reproductive autonomy was being stripped away. The combined effect was Theme 4 (‘Worried Women and Marginalised Mothers’), where women reported equal measures of worry and anger towards the system, society, and their situation and felt they had to reclaim agency, by making demands for information, care, and explanations during their perinatal journeys, which were blighted by restrictions, whilst the remainder of the population was allowed to live freely, without restriction. However, the back-drop to all of this was Theme 1 (‘A Failing System, Failing Women’), whereby the maternity care system was seen to be failing, which, in turn, was seen as a cause of all these other negative perinatal experiences, albeit exacerbated by the disparity between ‘Freedom Day’ outside, but not inside, maternity care. Taken together, the themes can be summarised as a theory of this specific population (women), phenomenon (pregnancy and postpartum period), and context (post-pandemic ‘Freedom Day’ in the UK), as ‘Freedom for some, but not for Mum’ (see Figure 2).

## 4 Discussion

New motherhood already presents as a major lifecourse transition. Combined with the unique pressures of the pandemic, perinatal women were at a heightened risk of elevated psychological stress (55, 56). Perinatal women were disproportionately affected by the COVID-19-related lockdown restrictions, resulting in increased feelings of anxiety and stress, fears surrounding the virus and its effect on their baby, lack of social support, and reduced accessibility and quality of maternity care services (19, 49). In particular, the persisting vulnerabilities, socio-economic and structural disadvantage and discrimination faced by many women of social disadvantage and ethnic diversity (57), may have been exacerbated during the pandemic which, once again, seemingly foregrounded issues of race and ethnicity within the healthcare system (39). Considering fair, high-quality maternity care is not a current reality, combined with the reduced visits, face-to-face contact, choices, continuity of care, delay in emergency procedures, closure of community centres, and the effect of self-isolation on mental health under lockdown, these inequalities have been intensified (58). The findings from this study detail how perinatal women perceived they were prioritised during the pandemic. They felt that care and support were sub-optimal and that restrictions were draconian, arbitrarily applied, and out of step with evidence-based guidance.

In and of itself, this is not a new finding from research into maternity care during the pandemic (59, 60), as numerous British reports have documented: safety concerns or poor care (7, 19, 61), including for perinatal mental health (24, 28, 48, 49, 62, 63); inadequate support from healthcare professionals (6, 47); blanket bans on chosen and consented birthing partners being present during antenatal care appointments and birth (9, 64, 65); and care lacking dignity and compassion when parents face a perinatal bereavement (20, 66). However, what is novel about this analysis is the perceived sense of reproductive injustice by perinatal women following the so-called ‘Freedom Day’, at which time the rest of the United Kingdom was once again allowed to enjoy ‘normal’ life. Maternity services remained places of enforced restrictions, diminishing women’s psychosocial wellbeing, and, ultimately, generating the feeling that they had been left behind. One area which may be unique to the circumstances of the pandemic is the issue of vaccination for perinatal women. Turning to syntheses of the pandemic literature [see (59, 60)], the lack of information about the effect of the COVID-19 virus on pregnancy and newborns and the efficacy and safety of vaccines has been raised as a particular issue. This was true of women of reproductive age in general (31, 32) as well as those going through their perinatal journeys (33). Given the official guidance on vaccination during pregnancy and whilst breastfeeding changed throughout the pandemic as new evidence became available to policymakers (3), it is possible women were conflicted about engaging in activities attributed to ‘Freedom Day’ whilst potentially remaining unvaccinated themselves. This therefore may mark a ‘triple burden’ experienced by perinatal women during the post-‘Freedom Day’ epoch. This ‘triple burden’ can be explained as follows: Firstly, perinatal women were burdened by the fear of being pregnant or postpartum during the pandemic and wanting to protect themselves against everyone else who has been unburdened by the ‘Freedom Day’ unlocking of restrictions (48, 49). Secondly, perinatal women were burdened by a restricted maternity care system which they had to navigate as there was no way of delaying their maternity care—unlike with elective procedures and routine operations (7). Thirdly, perinatal women were burdened by their decision-making processes as to whether or not they should be vaccinated against COVID-19, in the light of the changeable guidance across the pandemic lifecourse (32). Within this, we must also concern ourselves with the intersectional issues which accompany the burdens women in healthcare may face, such as being part of a minority ethnic group (57) or not having fluency in the language of the policy of care provided (67, 68), being a sexual minority (69), or facing additional health-related risks such as chronic illness (70). We must be cognisant, therefore, of how policy-level decision-making or the effects associated with implementing new policy (5) may disproportionately affect perinatal women, and not only monitor, but also take affirmative action (defined by Zohny et al. (71), p. 971 as: *‘A policy that ultimately aims at reasonably increasing the representation of minorities in the relevant area or reasonably addressing the disadvantages they suffer in the relevant area’.*) to reasonably adjust for women accessing antenatal, intrapartum, and postnatal healthcare.

Whilst the Government restrictions were enforced to protect the public, their removal for the general public after ‘Freedom Day’ arguably had a devastating effect on perinatal women. These women still faced restrictions in their care which removed them from their support networks (e.g., family, friends, antenatal, and mother-and-baby classes, and other community groups) which they held in high regard and as essential to their psychosocial wellbeing. Whilst the rest of the United Kingdom returned to a ‘new normal’ (72) which included regular socialisation, perinatal women were hindered from forming vital routines, social support networks, and friendships that may have assisted their maternity journey and protected their perinatal mental health (25, 28, 48, 49, 69).

In addition, ‘Freedom Day’ generated this feeling from harm being caused by the state of difference it created between perinatal women and the general public, but also perinatal women and other people seeking healthcare. Much like Wilkinson and Pickett (73) describe the worst social harm being derived not from poverty, but rather from inequality and the disparity of wealth being encountered and witnessed, the state of difference instituted by the so-called ‘Freedom Day’ made perinatal women see themselves as disadvantaged compared with the rest of the population. This made these women question why they had to receive care in particular ways (i.e., why was in-person care continuing to be sacrificed, in favour of virtual care); why social and physical distancing restrictions were still being enforced (meaning women had no agency over mode and place of birth, or who they had present with them); and why the information they were receiving was vague, differed depending according to source, and not forthcoming from official sources (including about vaccination and contracting the virus). Fundamentally, it made them ask why their care had not been reset in the same way as the rest of society (74). Even when women explained that they understood how maternity services were over-stretched and under-resourced (5, 36), this feeling of being so starkly contrasted against the general public enjoying post-‘Freedom Day’ normalities, often induced frustration at and with the healthcare system, and further inhibited the development of trustworthy relationships between perinatal women and their attending healthcare professionals. This was particularly true if healthcare professionals demonstrated incivility [see (39, 66)], especially when they were forced to suffer a perinatal bereavement or traumatic birth alone (9, 20, 66, 75).

Whilst the participants in this study did not discuss their personal experiences of social complexity, they did allude to the fact that these negative experiences would probably be more difficult amongst those women who find healthcare hard to access or have greater socio-demographic risks. This appears to be sensible, in-line with findings and commentary from both pre-pandemic (76) and para-pandemic (58) circumstances. However, there was a sole glimmer of hope within these data, in that the participating perinatal women discussed their reclamation of reproductive agency, turning their frustration and despair at the obvious disparity between post-‘Freedom Day’ society and still-restricted maternity services, into confident authority to challenge, demand answers, and advocate for themselves and their babies, at times where they struggled to navigate their own maternity care. In so doing, women in this study appeared to be reclaiming their maternity experiences, closing the gap on the disparity between themselves and the public enjoying the newly legislated freedoms, and ultimately subverting the reproductive injustices they faced in post-‘Freedom Day’ Britain.

### 4.1 Strengths, limitations, and future directions

The data collected for this study present a unique insight into a specific epoch of time, during which, despite the pandemic continuing across the World, the UK had removed all pandemic-related restrictions in public life. This presented a unique opportunity to collect data of such a particular zeitgeist, and the fact that both pregnant and postpartum women engaged in the research resulted in a richer understanding of the maternity experiences following ‘Freedom Day’. We are cognisant of the study’s limitations, such as the participants being skewed to a higher level of education than the general public, but we also recognise this as being a common issue with research which recruits via on-line survey or social media techniques. In addition, we note that all bar one of our participants were employed, which may have influenced how much time and resources women could deploy in navigating their care pathways. Whilst we achieved established parameters for saturation in grounded theory analysis, we surmise our relatively small sample could have been a result of participation exhaustion at time of data collection (77), which may have hindered recruitment somewhat, especially given the chronicity of the pandemic restrictions and circumstances.

Future research should consider issues of digital poverty or selection bias, and co-design specific strategies for recruiting women from, visible minorities, and lower socio-economic and educational backgrounds, to determine whether or not these findings are reproducible and, therefore, broadly generalisable, which we cannot accurately determine in this study, given the demographic make-up of our participants. Another priority should be to determine the impacts of these prolonged post-Freedom Day modifications to the provision of care on maternal, foetal, and newborn outcomes. Finally, we must synthesise and understand what evidence exists about persistent misinformation about COVID-19 vaccination in pregnancy, in order to address this and the associated loss of trust, and learn from it to enable improved messaging in the future pandemics.

## 5 Conclusion

This study uniquely documents the experiences of perinatal women in post-‘Freedom Day’ Britain, where all legal restrictions relating to social and physical distancing and other pandemic-related precautions were removed for the general public. Given that we know lifecourse adversity can affect later-life outcomes, both to physical and psychological health—our lifecourse approach suggests that this period of transition, which was marked by the pandemic lifecourse rupture, may lead these women to a lifecourse trajectory negatively affected by their experiences. These experiences might include but might not be limited to issues with bonding with their baby; relationship problems; future anxieties surrounding pregnancy and childbirth; and/or fear or distrust in the maternity healthcare policy and provision. As with studies of pregnant and postpartum women throughout the pandemic, these women experienced sub-optimal care; confusing information and messaging (especially with regard to the effect of the virus during pregnancy and vaccination against COVID-19); incivility expressed by staff who were clearly under-resourced and chronically fatigued by working in pandemic circumstances; and poor birthing experiences, often alone and not in-line with their preferences. When making policy to change perinatal healthcare availability and the way in which it is provided (especially in the light of health system shocks), it is crucial we are cognisant of how burden may be compounded amongst perinatal women and ensure we are making reasonable adjustments through affirmative action to reduce the negative consequences of reconfiguring healthcare and assimilating perinatal women into wider populations and daily life. What was distinctive with the women in this study was the disparity they witnessed and endured between general society living free of restrictions in a post-‘Freedom Day’ world, whilst they continued to utilise perinatal healthcare services which remained restricted. The moral harm caused by this state of difference led to defiance and ultimately compelled women to advocate for themselves, disrupting the ‘free-restricted’ dyad, and to demand better information, higher-quality care, and more agency during pregnancy and postpartum, all the whilst subverting the reproductive injustices they deemed themselves to have so unfairly endured.

## Data availability statement

The raw data supporting the conclusions of this article will be made available by the authors, without undue reservation.

## Ethics statement

The studies involving humans were approved by the University of Liverpool Research Ethics Committee. The studies were conducted in accordance with the local legislation and institutional requirements. The participants provided their written informed consent to participate in this study.

## Author contributions

SAS: Conceptualization, Data curation, Formal analysis, Investigation, Methodology, Project administration, Resources, Supervision, Validation, Visualization, Writing – original draft. EJH: Conceptualization, Data curation, Formal analysis, Methodology, Writing – original draft. LJ: Conceptualization, Data curation, Formal analysis, Software, Supervision, Writing – original draft. VF: Conceptualization, Project administration, Resources, Supervision, Writing – review & editing. AE: Supervision, Writing – review & editing. PvD: Supervision, Writing – review & editing. DJ: Supervision, Writing – review & editing. LAM: Supervision, Writing – review & editing.

## Group members of The Pregnancy and Motherhood [PRaM] during COVID Study Group

Laura K. Soulsby (orcid.org/0000-0001-9071-8654), University of Liverpool, UK; Paul Christiansen (orcid.org/0000-0001-7534-0948), University of Liverpool, UK; Leonardo De Pascalis (orcid.org/0000-0002-9150-3468), University of Liverpool, UK; and Joanne A. Harrold (orcid.org/0000-0002-0899-4586), University of Liverpool, UK.
